# INTERGENERATIONAL PLAY AND LEARNING: OPPORTUNITIES FOR ENGAGING PRESCHOOLERS, COLLEGE STUDENTS, AND OLDER ADULTS

**DOI:** 10.1093/geroni/igae098.1642

**Published:** 2024-12-31

**Authors:** Katherine Kwong, Monica Franzone

**Affiliations:** Connecticut College, New London, Connecticut, United States; Connecticut Collge, New London, Connecticut, United States

## Abstract

The U.S. Census notes that the population age demographic is rapidly changing, with approximately 10,000 individuals a day crossing the age threshold of 65, the mismatch between the number of individuals over the age of 65 and those under the age of 65 will have lasting implications in the coming decades. So, how can we take advantage of this resource? How can we benefit multiple generations? Intergenerational Play & Learning was a new course offered at Connecticut College during the Spring 2024 semester that introduced students to the intergenerational (IG) studies field and engaged them in experiential learning activities with older adults and preschool children. These students studied the changing relationships between young children and older adults through readings, videos, and personal observations. Through journals, interviews, and observation, students gained insights into the benefits of intergenerational learning (IL) and how IL can challenge discrimination. Course participants designed activities to promote social ties across age groups. The main goals for the course were: (1) provide a venue for young children, young adults, and older adults in the New London area to learn from and engage with each other; (2) encourage young and older adult participants to reevaluate stereotypes and discover how generations different from one’s own can provide an opportunity for learning; (3) evaluate the importance of family and community ties; and (4) develop a model for future intergenerational programming.

